# The effect of caponization on bone homeostasis of crossbred roosters. I. Analysis of tibia bone mineralization, densitometric, osteometric, geometric and biomechanical properties

**DOI:** 10.1038/s41598-023-41806-x

**Published:** 2023-09-04

**Authors:** J. Wojciechowska-Puchałka, J. Calik, J. Krawczyk, J. Obrzut, E. Tomaszewska, S. Muszyński, D. Wojtysiak

**Affiliations:** 1 Department of Animal Reproduction, Anatomy and Genomics, University of Agriculture in Kraków, 24/28 Mickiewicza Ave., 30-059 Cracow, Poland; 2 Department of Poultry Breeding, National Research Institute of Animal Production, 32-083 Balice, Poland; 3 Department of Animal Physiology, Faculty of Veterinary Medicine, University of Life Sciences in Lublin, 12 Akademicka St., 20-950 Lublin, Poland; 4 Department of Biophysics, Faculty of Environmental Biology, University of Life Sciences in Lublin, 13 Akademicka St., 20-950 Lublin, Poland; 5 Department of Animal Genetics, Breeding and Ethology, Faculty of Animal Sciences, University of Agriculture in Kraków, 24/28 Mickiewicza Ave., 30-059 Cracow, Poland

**Keywords:** Biological techniques, Developmental biology, Zoology

## Abstract

The presented study focuses on assessing the effect of caponization on the densitometric, osteometric, geometric and biomechanical parameters of tibial bones in crossbred chickens. The study was carried out on 96 hybrids between Yellowleg Partridge hens (Ż-33) and Rhode Island Red cockerels (R-11) aged 16 weeks, 20 weeks and 24 weeks. Birds were randomly assigned to 2 groups-the control group (n = 48; which consisted of intact roosters) and the experimental group (n = 48, which consisted of individuals subjected to caponization at the age of 8 weeks). The caponization had no effect on the densitometric, osteometric and geometric parameters (except the horizontal internal diameter of 16-week-old individuals) of tibia bone, as well as the content of calcium (Ca), phosphorus (P) and the Ca/P ratio in the bone mineral fraction in all analyzed age groups of animals. However, it contributes to a lower percentage of ash in the bones of capons at 20 and 24 weeks of age compared to cockerels. On the contrary, some mechanical and material parameters show the negative effect of caponization. Ultimate load and bending moment decreased in capons in all of the analyzed age groups of animals and yield load, stiffness and ultimate stress also decreased but only in the group of 20-week-old and 24-week-old individuals. This can contribute to the weakening of the capon bones, and in the perspective of prolonged maintenance to their deformation and even fracture.

## Introduction

The skeletal system, with the participation of skeletal muscles, forms the structural basis of support and locomotor activities, and it also performs protective functions for organs and tissues. Bones are made up of specialized tissue with a complex system of metabolically active cells and extracellular matrix that are necessary to maintain its structure. Genetic conditions, age, sex, nutritional and hormonal factors directly or indirectly affect the dynamics of ossification processes^1^. The proper functioning of the skeletal system affects the growth, development and metabolism of the entire organism and plays an important role in the effective breeding and rearing of poultry, being one of the main factors limiting the profitability of poultry production^2^. Changes in the skeletal system of poultry, often of unexplained etiopathogenesis, mainly lead to deformities of the bones of the hind legs. The birds' rapidly increasing body weight, as well as the imbalance in the growth of muscle mass relative to bone weight, significantly increase the risk of bone deformities and fractures, particularly of the tibia bone. Importantly, metabolic diseases of the leg bones result in lower daily weight gains, reduced slaughter weights, poorer feed utilization and consequently increased flock mortality^3,4^. A higher ratio of muscle to bone in poultry leads to disorders of growth and mineralization of the skeleton and the occurrence of various disease syndromes that reduce the quantity and quality of the products obtained while reducing production efficiency^5^. This reflects the insufficient adaptation of the birds' skeleton to the high body weight obtained in such a short period of time^2^. Moreover, financial losses due to the skeletal pathology in intensive flocks can be very often observed and they reach several hundreds millions of dollars a year^1,6^.

Currently, poultry livestock production is developing very intensively. Poultry kept in an intensive system is increasingly perceived as a product that is admittedly cheap, however of low quality. Therefore, the consumer market is increasingly looking for exclusive raw materials, distinguished by desirable sensory qualities of high standard. Products from so-called organic farming are particularly popular. Animal production of this type is based on free-range housing with a long rearing period and feeding on the farming feed. Native breeds are best suited for breeding in this type of system, due to genotypically and productionally diversity and which provide products with specific taste and quality^7–9^. Poultry production for laying is associated with obtaining unnecessary cockerels, the management of which is a considerable difficulty for the farm^10^. Taking into account the consumer tastes and firmly rooted culinary traditions, medium-heavy cockerels would be reasonable to be used in capon production.

A capon is a rooster that undergoes a castration procedure before reaching sexual maturity. This procedure is performed mainly to increase the weight of the animals and to improve the taste and dietary qualities of the meat. The minimum fattening period for these animals should be at least 11 weeks^8^. Under European conditions, the rooster’s testicles and epididymides surgical removal is considered the best method of caponization regardless of the origin of the birds^11–13^. For the production of capons in Poland, the most common are native breeds, i.e. Greenleg Partridge, Yellowleg Partridge, Polbar or Leghorn^14–16^. However, the small size achieved by these breeds has induced crossbreeding with the goal of obtaining a hybrid that achieves larger body size, while maintaining the unique taste and quality typical of native breeds^17,18^. Capon production is most popular in Asian countries, i.e. Taiwan^7,9,19,20^, China^21,22^ and in European countries including Spain, Portugal, Italy and France^23–26^.

Interference in the body's hormonal balance through castration surgery significantly affects the growth and development of individual organs and tissues. The loss of testicles results in a deficiency of androgens responsible for the development of physical and mental characteristics of males. As a result of this treatment, motility and aggressiveness among young cockerels decreases (reduction of fighting), and their comb recedes and becomes paler and more flabby^27–29^. Castration affects metabolic processes reflected in tissue structure, including the bone structure. The proper functioning of the skeletal system is determined by a number of factors including androgens. They regulate bone remodeling processes by affecting androgen receptors found in osteoblasts, osteoclasts and osteocytes. They also enhance bone formation by stimulating osteoblasts responsible for mineralization and bone matrix synthesis. In the available literature, there is little data on the effect of castration of cockerels on the processes of skeletal development, its growth and mineralization in connection with the development of tibia bone strength properties, and few papers deal with native breeds^30,31^. Thus, Muszyński et al.^30^ determined densitometric, geometric, structural and material characteristics of the tibia and femur bones of 24-week-old Polbar capons and roosters. In investigations conducted by Kwiecień et al.^31^, the physical, geometric, mechanical properties of femur and tibia of 24-week-old Polbar and Greenleg Partridge were measured. Similarly, Chen et al.^20^ examined the tibia bone characteristics in 26-week-old Taiwan country capons and cockerels. Although there is a lack of studies in the available literature on the effect of caponization on bone microstructure, it is worth noting that research on bone histomorphometry was conducted on various species of birds^81,82^. However, there is also no information on bone mechanics with regard to crossbreed hybrids. Hence, we hypothesized that the castration procedure impairs the development of the tibiofemoral bone in terms of its geometry and strength and that it also affects the mineralisation of the bone within the shaft making it more susceptible to deformation. Accordingly, this study was designed to evaluate the effect of caponization on tibia bone mineralization, densitometric, structural, geometric and biomechanical parameters.

## Results

### Body mass, osteometric and geometric parameters

**Figure 1:**
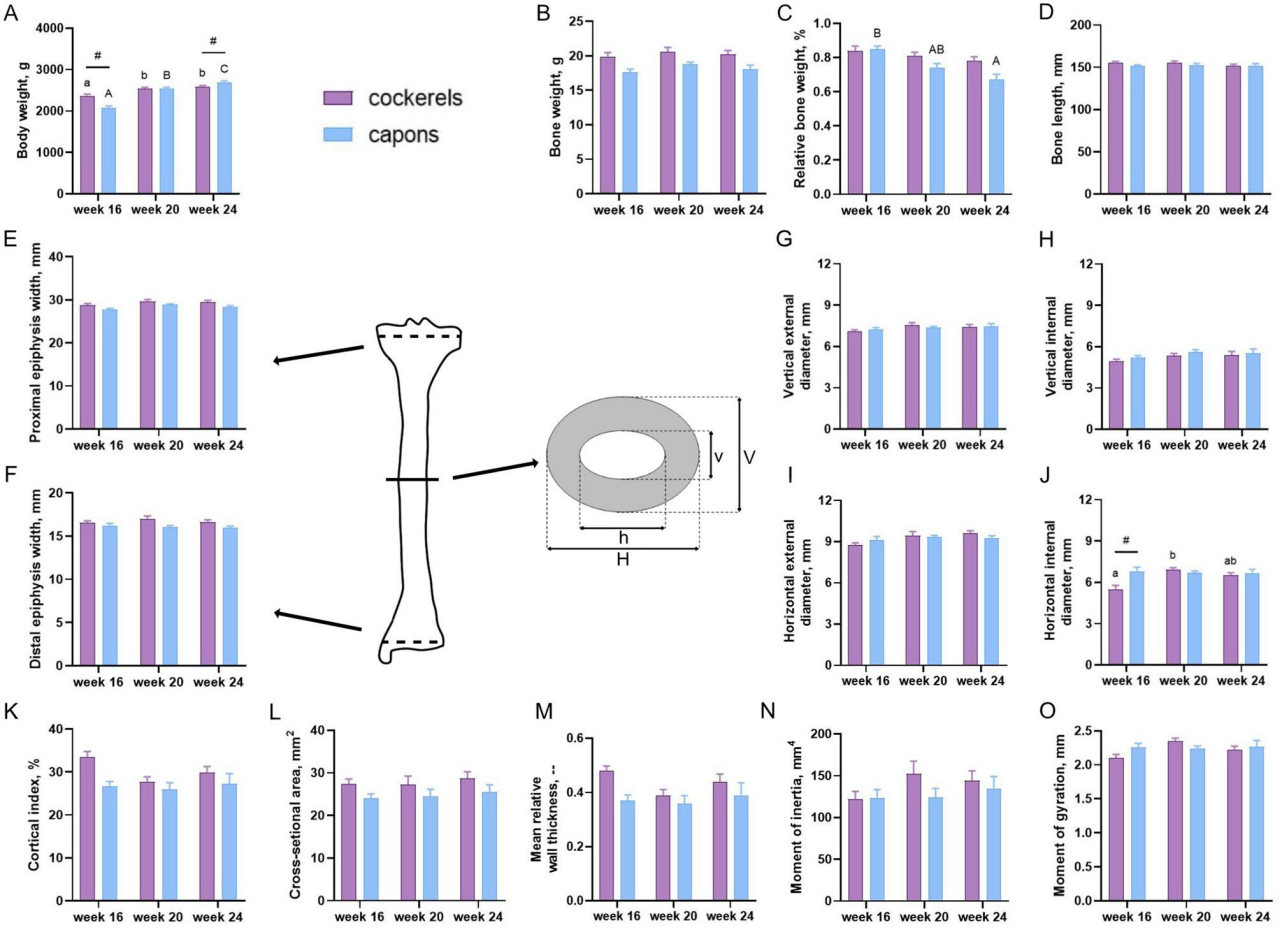
Body weight (**A**), osteometric and geometric parameters: bone weight (**B**), relative bone weight (**C**), bone length (**D**), proximal epiphysis width (**E**), distal epiphysis width (**F**), vertical external diameter (**G**), vertical internal diameter (**H**), horizontal external diameter (**I**), horizontal internal diameter (**J**), cortical index (**K**), cross-sectional area (**L**), mean relative wall thickness (**M**), moment of inertia (**N**), moment of gyration (**O**) of tibia bone of 16-week-old, 20-week-old and 24-week-old cockerels and capons and measurements of external and internal diameters in the horizontal and vertical planes of bone cross-section. Data are presented as least squares means (LSM) and standard error of mean (SE), a, b, c-mean values between age groups within cockerels with different letters differ significantly *P* < 0.05; A, B, C-mean values between age groups within capons with different letters differ significantly *P* < 0.05; #-significant difference between capons and cockerels within age (*P* < 0.05).

Capons in the 16th week of life were characterized with lower body weight compared to cockerels. On the contrary, 24-week-old capons had significantly higher body weight compared to cockerels. In the group of 20-week-old animals, body weight did not differ between control and experimental group (Fig. 1A). Moreover, caponization did not influence the bone weight, relative bone weight, bone length, proximal and distal epiphysis width, horizontal external diameter H, vertical external diameter V, vertical internal diameter v, cortical index CI, cross sectional area A, mean relative wall thickness MRWT, moment of inertia Ix and moment of gyration Rg (Fig. 1B–O). Among all of determined osteometric and geometric parameters only horizontal internal diameter h significantly increased in the 16-week-old capons, as compared to those of the cockerels. Such relationships were not noticed in the case of animals in the 20th and 24th week of their rearing period (Fig. 1J).

### Bone densitometric parameters

**Figure 2:**
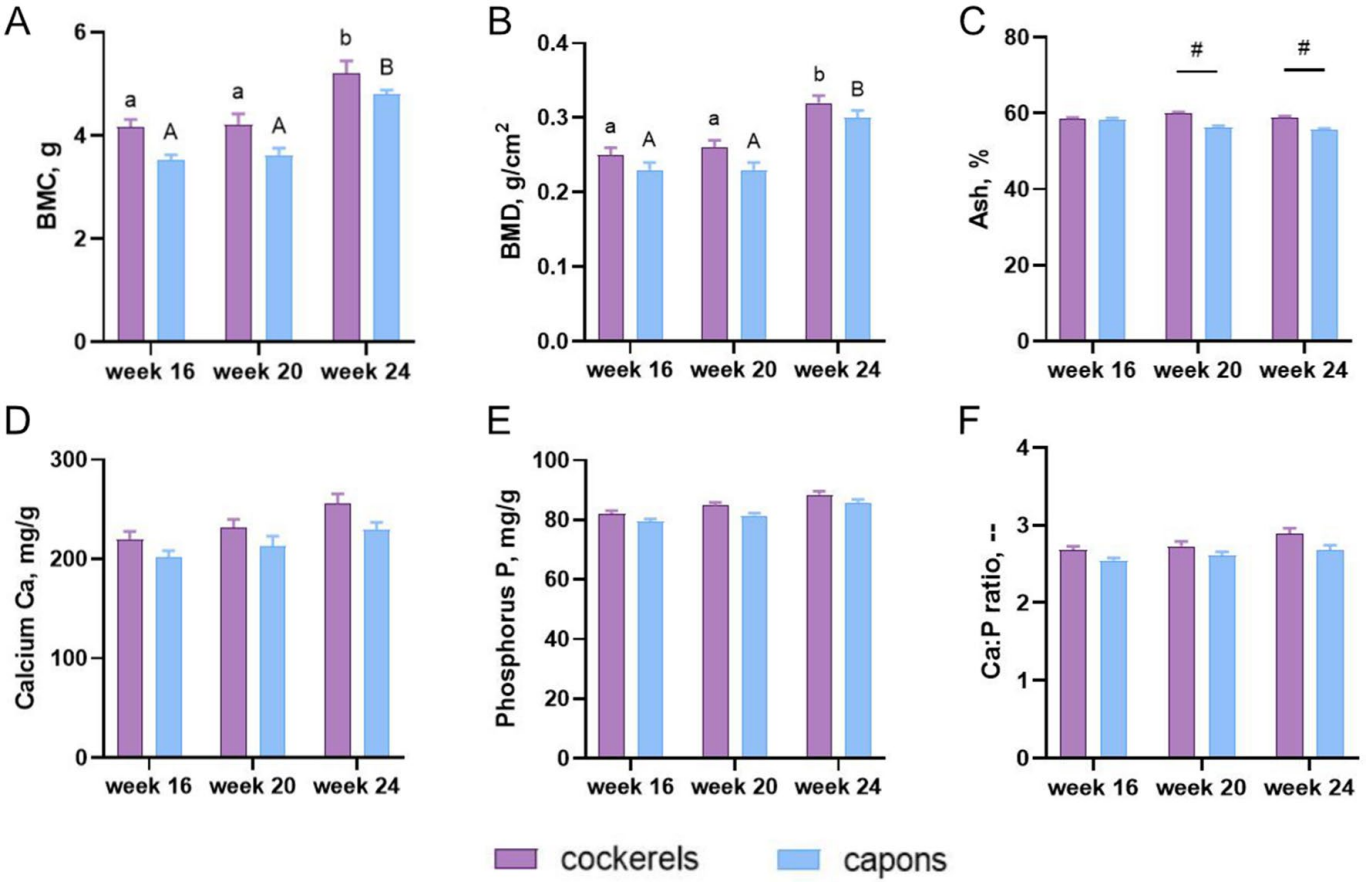
Densitometric parameters: bone mineral content (BMC) (**A**), bone mineral density (BMD) (**B**) and bone mineral composition: ash (**C**), calcium (**D**), phosphorus (**E**), Ca:P ratio (**F**) of tibia bone of 16-week-old, 20-week-old and 24-week-old cockerels and capons. Data are presented as least squares means (LSM) and standard error of mean (SE), a, b, c-mean values between age groups within cockerels with different letters differ significantly *P* < 0.05; A, B, C-mean values between age groups within capons with different letters differ significantly *P* < 0.05; #-significant difference between capons and cockerels within age (*P* < 0.05).

The results of analysis of measurements of densitometric parameters, i.e. BMD and BMC in the mid-diaphyseal region of the tibia bone of cockerels and capons at 16, 20 and 24 weeks of age are presented in Fig. 2A,B. There were no caponization effects on BMD and BMC in all of the analyzed age groups of birds (Fig. 2A,B).

### Ca, P and ash content in bone

The analysis of mineral composition showed that capons bones at 20 and 24 weeks of age had a significantly lower percentage of ash, compared to the cockerels (Fig. 2C). On the contrary, caponization did not influence the ratio Ca:P, Ca and P content (Fig. 2D–F).

### Mechanical parameters

**Figure 3:**
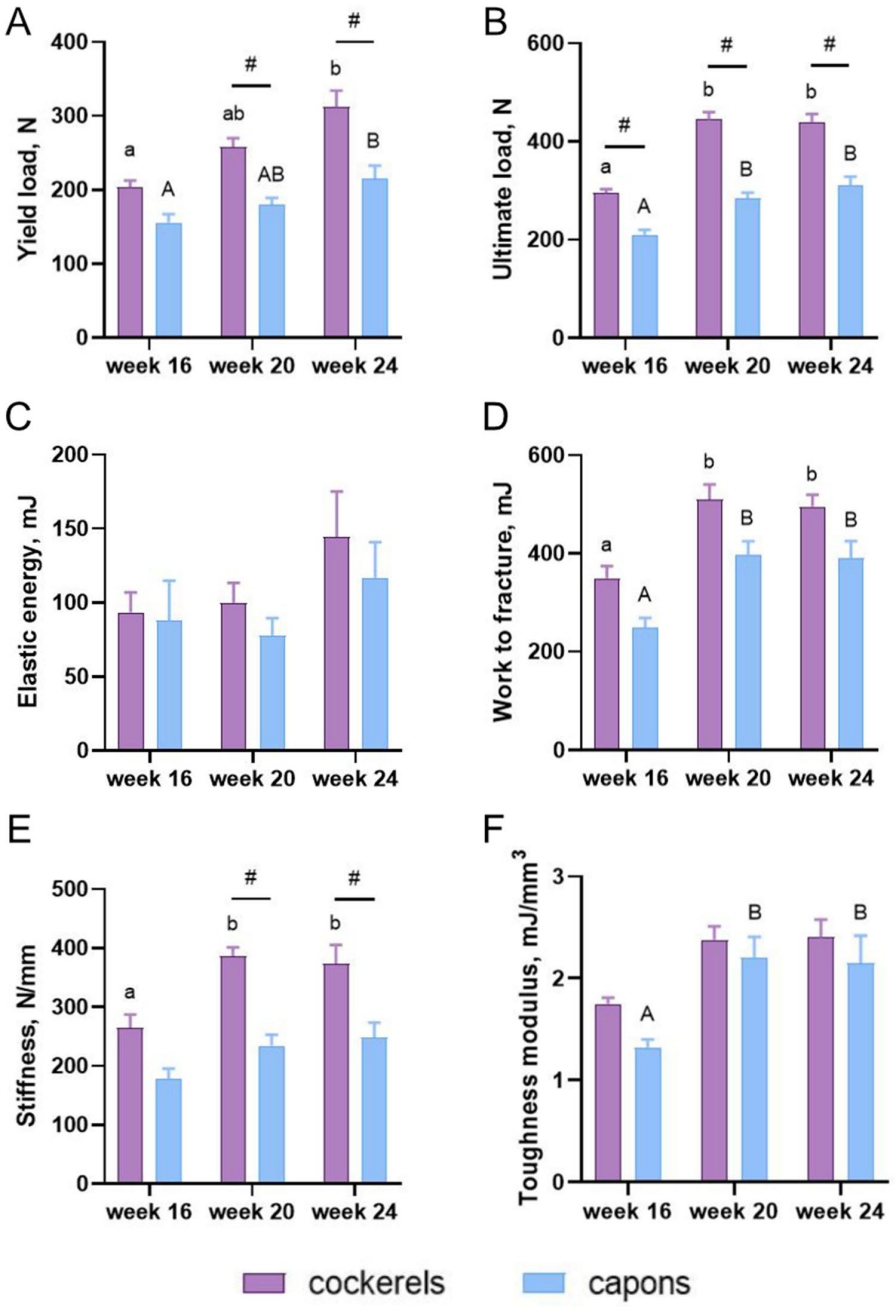
Mechanical parameters: yield load (**A**), ultimate load (**B**), elastic energy (**C**), work to fracture (**D**), stiffness (**E**), toughness modulus (**F**) of tibia bone of 16-week-old, 20-week-old and 24-week-old cockerels and capons. Data are presented as least squares means (LSM) and standard error of mean (SE), a, b, c—mean values between age groups within cockerels with different letters differ significantly *P* < 0.05; A, B, C-mean values between age groups within capons with different letters differ significantly *P* < 0.05; #-significant difference between capons and cockerels within age (*P* < 0.05).

The caponization affected the decrease of ultimate load F_max_ among all analyzed age groups of animals (Fig. 3B). Furthermore, both the yield load F_el_ and stiffness S decreased in the capons compared to the cockerels, but only in the group of 20-week-old and 24-week-old individuals (Fig. 3A and E). In contrast, the value of elastic energy W_el_, work to fracture W_max_ and toughness modulus u were not influenced by the castration (Fig. 3C,D,F).

### Material parameters

**Figure 4:**
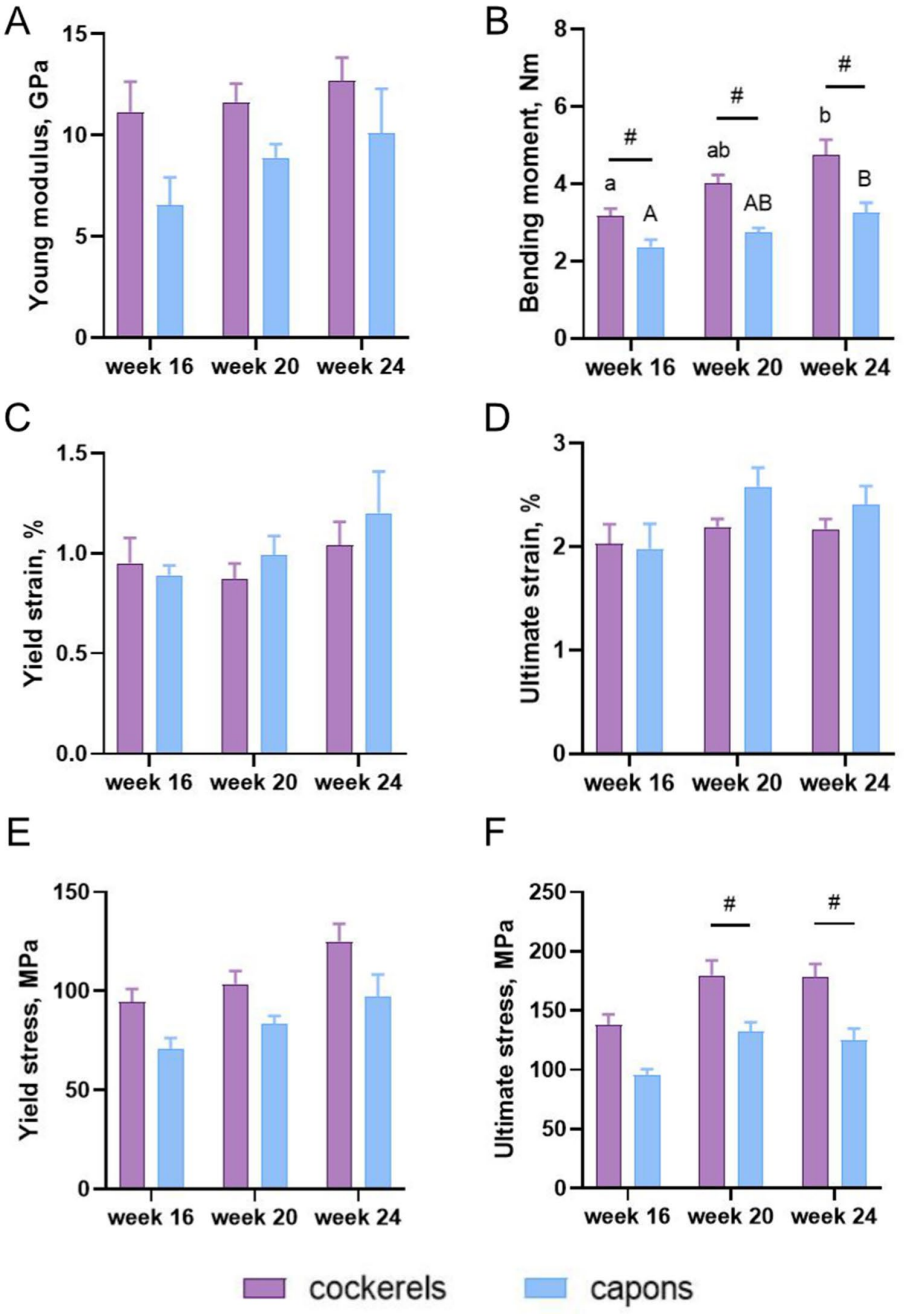
Material parameters: Young modulus (**A**), bending moment (**B**), yield strain (**C**), ultimate strain (**D**), yield stress (**E**), ultimate stress (**F**) of tibia bone of 16-week-old, 20-week-old and 24-week-old cockerels and capons. Data are presented as least squares means (LSM) and standard error of mean (SE), a, b, c-mean values between age groups within cockerels with different letters differ significantly *P* < 0.05; A, B, C-mean values between age groups within capons with different letters differ significantly *P* < 0.05; #-significant difference between capons and cockerels within age (*P* < 0.05).

The castration resulted in lower values of bending moment M in the experimental group, compared to the control group in all analyzed rearing period groups of animals (Fig. 4B). Similarly, in the case of ultimate stress σ_f_, caponization decreased value of this parameter, but only in groups of individuals at 20 and 24 weeks of age (Fig. 4F). Castration did not influence the Young modulus E, yield strain ε_y_, ultimate strain ε_f_, yield stress σ_y_ (Fig. 4A,C,D,E).

## Discussion

During the growth and maturation, steroid sex hormones including testosterone play an important role in regulating bone metabolism. Androgens have an inhibitory effect on the osteoclastic bone resorption and they are necessary for achieving and maintaining normal bone and shape among males^32–34^. In addition, they stimulate protein synthesis to increase muscle mass and affect the deposition of calcium in the bones. Moreover, testosterone deficiency results in the inhibition of the development of mental and physical characteristics of cockerels^21,28,35^. As indicated by earlier studies, caponization influenced the weight gain and increased pectoral and leg muscle mass^19,36,37^, which is undoubtedly beneficial for their breeding. On the other hand, an imbalance in the ratio of muscle mass to bone mass can lead to excessive overload, resulting in bone deformities and even in fractures.

Our study showed that caponization had no effect on body weight in a group of 20-week-old individuals. These results correspond with several other studies^25,36,38^. Moreover, in presented study a drop in body weight in capons, compared to cockerels was noticed in the group of 16-week-old animals, and was also observed in earlier study^21^. In contrast, our study also showed that 24-week-old capons achieved significantly higher body weight compared to cockerels. Similar results were observed in a different study^30,31,37^. The differences in body weight of capons, shown in many studies, are likely due to the use of various breeds, lines or crossbred, as well as differences in the duration of rearing, different feeding and the age of the birds at which the castration procedure was performed^39^.

An important criterion for evaluating bone abnormalities is the measurement of bone density. To determine bone quality and risk of bone fracture are used densitometric methods such as dual-energy X-ray absorptiometry DXA. Numerous studies show that androgen deficiency reduces bone mineral density in both humans and other mammals^40–43^. On the contrary, in the case of birds, the results of studies are not conclusive. As reported in Zawacka et al.^44^ in the study conducted on individuals at 12-week-old and 24-week-old Polish native breed, Greenleg Partridge, they observed higher BMD values within combined compact and cancellous bone of tibiotarsal bone in capons, compared to cockerels. Furthermore, Muszyński et al.^30^ determined BMD and BMC values for the whole bone and separately for proximal and distal heads and mid-diaphyseal part of the femur and tibia of 24-week-old Polbar cockerels and capons. They showed reduced values of BMD in the midshaft of both bones and in the proximal epiphysis of femur in capons, compared to cockerels. In turn, Tomaszewska et al.^54^ assessed tibia and femur densitometric parameters of total bone, midshaft and proximal and distal region of 24-week-old cockerels and capons of two breeds, i.e. Polbar and Greenleg Partridge. They found a reduction in BMD of total femoral bone in capons, compared to cockerels in both breeds. Additionally, in the case of Greenleg Partridge, they noted lower values of BMC of total and midshaft part of femur, as well as midshaft part of tibia in capons, compared to cockerels. Also, what it is important, authors did not observe a negative effect of caponization on the other densitometric parameter of tibia bone, which is consistent with present study, where no significant effect of castration on the BMD and BMC levels of tibia bone of 16-, 20- and 24-week-old individuals were observed. Taking some reports into account it was shown that androgen deficiency has led to decrease in bone mineral density and consequently osteoporotic changes in bone tissue^45,46^. It seems that factors such as breed, nutrition, age, living conditions or used testing methods also influence densitometric parameters.

The anatomical structure as well as the size of bone determines its mechanical strength^47,48^. The adaptation of bones to counteract pressures exerted on them occurs through variations in their shape and size and the internal structure of the bone tissue. Many studies analyzing changes in the mechanical strength of the avian skeletal system associated with individual growth have shown that the body weight of birds is mainly based on the tibia bone, therefore it is considered as a model bone in this type of studies^30,49^. Literature data indicate that steroid hormones are necessary for the proper metabolism of bone tissue, and as a result of their deficiency, pathological changes may occur within it^50,51^. Confirmation of these observations could be osteometric studies. Chen et al.^52^ in a study conducted on the 26-week-old Leghorn birds observed that caponization decreased tibia length. This result is in accordance with similar studies^35,44,53^. In the conducted research, it was noted that castration did not influence the tibia length in all analyzed rearing period groups of animals, which is consistent with other studies^7,20,30,31,54^. Varied results also apply to the bone mass. Negative effects of caponization on this parameter was reported by Chen et al.^20^ and Chen et al.^53^. A similar reduction in bone mass of tibia bone among capons, compared to cockerels was shown by Zawacka et al.^44^, but only among individuals slaughtered at 24 weeks of age, whereas in the case of 12-week-old animals authors found no such effect. However, similarly to the presented study, other studies report that caponization has no significant effect on tibia and femur bone mass^14,30,31,52,54,55^.

On the other hand, analyzing the effect of caponization on such parameters as: proximal and distal epiphysis width and relative bone weight of tibia bone, in our study these parameters were not influenced by the castration in all of the analyzed age groups of birds. The relative bone weight results included in this work are consistent with the previous reports^30,55^. Contrary to these however, were the results observed by Chen et al.^20^ and Chen et al.^52^, where the caponization decreased the relative bone weight. Similarly, Tomaszewska et al.^54^ and Kwiecień et al.^31^ showed the negative effect of castration on the value of this parameter for both femur and tibia bones. Furthermore, the results of geometric parameters of bones are ambiguous. In the experiment of Muszyński et al.^30^, they found that the tibia and femur midshaft of capons were characterized by larger diameters, compared to cockerels. Tomaszewska et al.^54^ found that caponization led to the increase of horizontal internal and external diameter and vertical external diameter of tibiotarsal bones of Polbar individuals. No effect of castration was observed in the case of either vertical internal diameter of Polbar and all diameters of tibia midshaft of Greenleg Partridge. Additionally, the same research shows higher values of horizontal external diameter and vertical internal and external diameter of femur bone of Polbar capons compared to cockerels. In the femur of Greenleg Partridge, the diameter dimension did not differ from those of cockerels. However, similar results were reported by Kwiecień et al.^31^. Our study showed no significant effect of castration on these parameters, except for the horizontal internal diameter of the 16-week-old individuals’ tibia bone, where significantly higher values were observed among capons compared to cockerels. The differences in the geometric parameters of the long bones of capons in relation to those of cockerels are observed in the literature. The values of these parameters are determined not only by castration, but also by the age, breed and types of bones. In this experiment, the lack of gonadal hormones among capons also did not affect the mean relative wall thickness MRWT and the cortical index CI of tibia bone, which is consistent with the results reported by Muszyński et al.^30^, Tomaszewska et al.^54^ and Kwiecień et al.^31^. However, in our study, different results were obtained for the cross sectional area A parameter. Muszyński et al.^30^ noted a larger cross sectional area of the tibia and femur of capons compared to cockerels. Similar alteration in the Polbar’ femur, has been observed by Tomaszewska et al.^54^. On the other hand, in the case of tibia bone of Polbar and Greenleg Partridge, the authors showed no effect of caponization on the value of this parameter. It is consistent with our study and other observations^31^. Only few of the scientific reports take into account the parameters, i.e. moment of inertia and moment of gyration, which are not a direct geometric parameter, but serve to determine its mechanical strength (material properties)^30,56^. Some researchers observed an increase in the moment of inertia and moment of gyration of the tibia and femur of capons compared to cockerels^30,31^. In contrast, in the present study, castration did not affect the moment of inertia and moment of gyration of tibia in all analyzed rearing period groups of animals. There are many indications that the differences in osteometric and geometric properties between capons and cockerels, observed in the literature, may be due to the use of different breeds, lines, rearing and feeding technologies.

Analysis of the bone’s supportive properties is possible by studying the strength parameters. One method of assessing them is the three-point bending test. It is known that bone strength is affected by many factors, one of which is sex steroid hormones. Hence, the analysis of hormone levels on the mechanical parameters of bone tissue has been the subject of numerous studies among both birds and mammals^9,58,59^. In presented work, caponization resulted in the reduction of the yield load F_el_ of the tibia bone only in the group of 20-week-old and 24-week-old birds. Another study has also shown no effect of caponization on yield load although, in contrast to our experiment, this study involved both femur and tibia bone^30^. Similarly, Tomaszewska et al.^54^ found a reduction in the value of this parameter in capons but only in the case of the femur of Greenleg Partridge birds. On the other hand, in the case of femur of Polbar and the tibia of Polbar and Greenleg Partridge individuals, authors did not observe differences in yield load between cocks and capons. Kwiecień et al.^31^ also showed a reduction in yield load in capons compared to cockerels, but only in the femur. In the case of the tibia bone, authors noted no effect of caponization on the value of this parameter, which is consistent with our results among 16-week-old individuals. The varied results of the study also apply to the ultimate load parameter. Muszyński et al.^30^ found no effect of caponization on the value of this parameter for both tibia and femur. Also, Kuźniacka et al.^60^ did not demonstrate the effect of castration on ultimate load of the tibia and femur of 16-, 18- and 20-week-old Plymouth Rock birds. In contrast, Tomaszewska et al.^54^ observed a reduced value of the ultimate strength of the femur of 24-week-old capons of Greenleg Partridge compared to cockerels of the same breed and age. No difference in ultimate load between cockerels and capons within the femur of Polbar individuals and tibia bone of both breeds were observed by the authors. Similarly, Kwiecień et al.^31^ showed that caponization affects the reduced value of this parameter in capons compared to cockerels, but only in the case of tibia bone. A number of studies also have shown that sex steroid hormone deficiency in capons leads to lower values of ultimate load of tibia bone compared to individuals with normal level of testosterone^20,52,53^, which is also consistent with the results of our study. Moreover, the available literature lacks information on the effects of caponization on mechanical strength properties, i.e. elastic energy, work to fracture, stiffness and toughness modulus. Only Muszyński et al.^30^ conducted this type of analysis, but excluding the toughness modulus. However, the authors did not demonstrate the effect of castration on the values of the above-mentioned parameters. No differences in elastic energy and work to fracture between capons and cocks were also found in the presented work. In contrast, stiffness (describing the bone resistance to deformation) in our experiment showed a reduced value among capons compared to cockerels, but only among 20-week-old and 24-week-old animals. Whereas in the group of birds slaughtered at the 16th week of age, there were no significant effects of caponization on the value of this trait. Martens et al.^61^ and Stromsoe et al.^48^ indicate that bone geometry has a significant effect on its properties during deflection. Hence, it can be assumed that changes in the mechanical parameters of bone may be related to changes in the geometry of the marrow cavity.

More complete information related to changes in the structure of bone tissue are provided by studies of the material parameters of bone. In our study, the castration had no significant effect on Young modulus in all of the analyzed age groups of birds. On the contrary, Muszyński et al.^30^ note that castration decreases Young modulus, for both femur and tibia bone. Moreover, the results of our experiment and previous studies demonstrated that bending moment M of bone decreased after caponization^30,53^. Research conducted on birds bred until the 16th, 18th and 20th week of age, showed that there were no differences between cocks and capons in terms of yield strain ε_y_ of tibia bone in all tested age groups of animals^60^, which is consistent with the results of our study. However, in another experimental trial^30^ an increase in the yield strain of femur and tibia bone of capons compared to cockerels were shown. It should be noted that bone fracture usually occurs when the learning of the „material” on the opposite side to the applied force happens. The deformation arising during this process is referred to as ultimate strain ε_f_. Hence, this indicator is very important to evaluating the material parameter of bones. In our study, as well as in Muszyński et al.^30^ work, no effect of caponization on the value of this parameter was observed. Confirmation of these results may also be provided by the observation of Kuźniacka et al.^60^, who reported no changes in deflection at the femur and tibia fractures between capons and cockerels at 18th and 20th weeks of age. Interesting information is also provided by Muszyński et al.^30^, which determined the effect of caponization on the yield stress σ_y_. The authors observed a reduced value of this parameter in both long bones (femur and tibia) among capons compared to cocks. On the other hand, in the presented work, caponization did not influence the yield stress σ_y_ in all analyzed rearing period groups of animals. On this basis, it can be concluded that stresses developed during deflection in the presented experiment, were similar in the bones of cockerels and capons, and this may indicate that their response was the same as the deformation. It has been speculated that the castration procedure may also affect the ultimate stress σ_f_ (maximum stress that a bone could hold off in bending before fracture) which is supported by reports by Lin and Hsu^7^, who observed a reduction value of this parameter in the tibia bone of the 28-week-old capons of Taiwan country compared to cockerels of the same age and breed. Similar results were obtained for tibia bone in the current study, but only among 20-week-old and 24-week-old individuals. Another studies has also shown reduced values of these parameters as a result of caponization^20,30,52^. On the other hand, Chen et al.^53^ found no effect of caponization on tibia ultimate stress of 40-week-old Taiwan country birds, which was also observed among 16-week-old individuals in the presented experiment. The discrepancies in the present study may indicate that caponization affects the deterioration of bone tissue in the tibia bone region, but does not interfere with its ability to perform its support-bearing function, probably due to adaptive changes in bone geometry.

One of the factors affecting the mechanical strength of bone is the degree of its mineralization^62,63^. According to Currey^64^ and Schaffler and Burr^65^ there is a relationship between mineralization and the elastic modulus of bone, which directly affects its behavior during elastic deformation. A high degree of mineralization increases the bone fragility and lowers the total energy required to break it. In turn, too much reduced mineralization increases the work to fracture bone and increases the deformability^66^. According to the literature, sex hormones play an important role in the mineralization of the skeletal system^67^. Tomaszewska et al.^54^ determined the ash content of long bones (tibiae and femora) of 24-week-old cockerels of the Polbar and Greenleg Partridge breed. They found a negative effect of the castration procedure only in the femur of Polbar birds. In the case of the tibia bone of Polbar capons and the tibia and femur of capons of Greenleg Partridge did not differ in the level percentage of bone ash compared to the cockerels. Similarly, Lin and Hsu^7^ showed no changes in the level of tibial ash of 28-week-old capons compared to cockerels of the same age. Chen et al.^55^ analyzing the tibial ash content of 26-week-old Leghorn capons, found lower levels of bone ash among capons compared to cockerels. Other authors have also proved reduced bone (femoral and tibia) mineralization in capons^30,31^. These observations also confirm the results obtained in the present study, which indicate reduced mineralization of the tibia bone as a result of caponization, but only among 20-week-old and 24-week-old individuals. In a group of 16-week-old birds, there was no effect of castration on the value of this parameter and this suggests that persistent testosterone deficiency as capons age slow down the rate of mineralization and mineral formation in the tibial bone matrix. The mineralization depends on the content of calcium Ca and phosphorus P in the bone and their molar ratio^68^. Previous studies indicate that lack of testosterone may contribute to the disruption of these macroelements levels in the bones^9,55^. Muszyński et al.^30^ analyzed the Ca content of the tibial and femoral bones of 24-week-old Polbar cockerels and capons. Authors showed that lack of sex steroids contributes to lower Ca level only in the femur. Similar results were obtained by Kwiecień et al.^31^. On the other hand, the negative effect of caponization on Ca levels in the tibia bone was shown by Lin and Hsu^7^ among the individuals at their 28th week of rearing period. Also, Lin et al.^9^ showed a reduction in Ca content in the tibia bone of capons at 26th and 30th week of age compared to cockerels of the same age. In the other groups (at the 14th, 18th, 22nd and 35th week of age), the authors noted no effect of castration on the Ca level. Another study has also shown no effect of caponization on the Ca content in the femur and tibia bone^54^. Similar results were obtained for the tibia bone in the presented experiment for all analyzed age groups of animals. These observations are also consistent with the results of Chen et al.^55^, where they also observed no effect of caponization on Ca content of tibia bone of Leghorn capons and cockerels at their 26th week of age. As mentioned earlier, P is an essential factor regulating bone mineralization. In the present study, the caponization did not cause changes in the P content of the tibia bones, which was also observed in previous investigations^7,31,54^. On the other hand, some reports noted different results. Muszyński et al.^30^ found that capons’ long bones (tibial and femoral) were characterized by lower level of P compared to cockerels’ bones. Moreover, similar results were reported by Chen et al.^55^. In contrast, Lin et al.^9^ observed a reduction in the P content of tibia bone of capons compared to cockerels, but only at the 18th, 26th and 30th week of their rearing period. In the case of other groups, at the 14th, 22nd and 35th week of age, the castration had no effect on the value of this parameter. It is not only the determination of Ca and P in the bones that is used to assess the mineral composition of the bones, but also their mutual ratio which is important. Similar to the presented experiment, most of the previous studies found no changes in the ratio of Ca:P in the tibial and femoral bones as a result of caponization^31,54^. On the other hand, Muszyński et al.^30^ noted an increased ratio of these macroelements, but only in the tibia bone of capons compared to cockerels.

In conclusion, our study has shown that caponization negatively affected the bone mineralization and some mechanical and material tibia bone parameters, especially among the 20-week-old and 24-week-old individuals. However, it is worth mentioning that this research has several limitations. There is a lack of dynamic histomorphometry analysis. Moreover, bone turnover markers in plasma were not measured. These issues require additional study.

## Material and methods

### Ethics approval

All animal procedures were approved by the 2nd Local Institutional Animal Care and Use Committee, Institute of Pharmacology, Polish Academy of Sciences in Krakow, Poland (No. 1121 of 27 November 2014). Experiment was performed accordingly to the European Union directive no. 2010/63/EU and with the appropriate ARRIVE guidelines for reporting on experiments involving animals.

### Animals and experimental groups

The study was performed at the National Research Institute of Animal Production of Balice n. Krakow. The experimental material comprised of 96 hybrids between Yellowleg Partridge hens (Ż-33) and Rhode Island Red roosters (R-11) from the conservation flock of the National Research Institute of Animal Production of Balice n. Krakow. The animals were randomly assigned to two groups-the control group (n = 48), which consisted of uncastrated cockerels and the experimental group (n = 48), which consisted of castrated birds in their 8th week of life. Both of these groups were slaughtered by decapitation at three different periods of their life-in the 16th, 20th and 24th week of their lives (EU Regulation No. 543/2008 of 16 June 2008 laying down detailed rules for the application of Council Regulation (EC) No. 1234/2007 as regards the marketing standards for poultry meat and EU Regulation No. 1099/2009 of 24 September on the protection of animals at the time of slaughtering). The caponization procedure was performed by a licensed veterinarian under local anaesthesia in their 8th week of life. All methods were performed in accordance with the relevant guidelines and regulations. Birds were kept in standard environmental conditions (temperature 16–18°C, relative humidity 60–75%) in the barn system with a stocking density of 7 birds/m^2^. During the entire period of rearing and fattening, i.e. 16th, 20th and 24th week of age, capons and cockerels were allowed free access to food and water. All animals were fed on a diet corresponding to the rearing periods (three-phase feeding): mixture I (1–7 weeks), mixture II (8–16 weeks) and mixture III (17–24 weeks). The results of nutrient analysis of feed materials according to the AOAC procedures are shown in Table 1. Eight individuals with a body weight close to the average in their group were selected for slaughter at the 16th, 20th and 24th week of their life. Cockerels and capons did not receive feed for about 12 h before slaughter, but they were provided with constant access to water. Immediately after slaughter, the effectiveness of the castration procedure was checked (removal of the testes). Many papers analyzing changes in the strength of the skeletal system of birds have shown that the body weight is mainly based on the tibia bone, so it is considered a model bone in this type of study. Therefore, right and left tibia bones were taken.

**Table 1:** Chemical analysis of the diet fed during the trial.

	Mixture I:1–7 weeks	Mixture II: 8–16 weeks	Mixture III: 17–24 weeks
Dry matter [%]	88.38	87.86	88.54
Crude ash [%]	7.37	5.83	3.96
Crude protein [%]	19.30	18.66	16.30
Crude fat [%]	2.23	2.02	2.29
Crude fibre [%]	2.34	2.54	2.38
Metabolizable energy [MJ/kg]	11.92	12.05	12.18
Metabolizable energy [kcal/kg]	2850	2880	2910

### Bone collection

Immediately after slaughter, right tibiae from individual birds were isolated, scraped away from any soft tissues and kept frozen at –25°C for further analysis.

### Reagents

Nitric acid was purchased from Sigma-Aldrich, St. Louis, MO, USA.

### Bone analysis

The bone weight and length, the width of proximal epiphysis, the width of distal epiphysis were measured using Adventurer Pro AV513CM electronic balance (Ohaus Europe GmbH, Nanikon, Switzerland) and STALCO s-11115 electronic caliper with an accuracy of 0.01 mm. Relative bone weight (%) was calculated as a ratio of bone weight to body weight as described previously^54,69^.

After the measurements of osteometric parameters, densitometric measurements of the bones were performed using the dual-energy X-ray absorptiometry (DXA) method on Discovery W densitometer (Hologic Inc., Bedford, MA, USA). Based on taken scans, bone mineral density (BMD) and bone mineral content (BMC) were determined. The analysis was performed for the mid-diaphyseal region according to the methodology proposed by Akhter et al.^70^ and Tomaszewska et al.^54^. Accordingly, the measurements in our work were performed on the scanned data using operator-defined regions of interest (ROIs) covering the mid-diaphyseal fragment of bone. All analyses were carried out by the same person (S.M.).

The analysis of the bone mechanical properties were determined based on the three-point bending test, using a Zwick Z010 universal testing machine (Zwick GmbH & Company KG, Ulm, Germany) connected to a recording computer with TestXpert II 3.1 software (Zwick GmbH & Company KG, Ulm, Germany). In our research a measuring head of an operation range of 10 kN was used. The bones were placed on supports with distance corresponding to 40% of the total bone length. The midshaft part of tibia bone was loaded in the anterior posterior (A-P) plane of bone at a displacement rate of 10 mm/min. Based on the obtained load–displacement curves, the following bone mechanical parameters were determined: yield load F_el_, ultimate load F_max_, elastic energy W_el_, work to fracture W_max_, stiffness S and toughness modulus u^30,71,72^. All mechanical parameters were determined using Origin 2016 software (Origin Lab, Northampton, MA, USA).

After the three-point bending test, the vertical (v, V) and horizontal (h, H) diameters (external and internal) were measured using STALCO s-11115 electronic caliper with an accuracy of 0.01 mm^73^. Scheme of the measurements of external and internal diameters in the horizontal and vertical planes of tibia is presented in Fig. 2. Based on these measurements, the following geometrical parameters of the tibia were calculated: mean relative wall thickness (MRWT), cortical cross-sectional area A, cortical index CI, second (cross-sectional) moment of inertia I_x_ and moment of gyration R_g_^30,71,74,75^.

On the basis of data collected during the three-point bending tests and measured geometric parameters, the following material properties of the bone were defined: Young modulus E, bending moment M, yield strain ε_y_, ultimate strain ε_f_, yield stress σ_y_, ultimate stress σ_f_^30,71,72,76^.

After evaluating mechanical parameters, the midshaft part of the tibia was defatted, dried at 105°C for 24 h to remove the bound water, and then cooled to room temperature in vacuum desiccator^77,78^. The tibia mid-diaphysis fragments were crushed in a porcelain mortar to a fine powder and weighed using WAX62 electronic balance (Radwag, Radom, Poland) with an accuracy of 0.0001 g. Finally, the samples were mineralized in a muffle furnace (Czylok, Jastrzebie Zdroj, Poland) at 500°C for 12 h to determine bone ash percentage. Analysis of the ash content was performed using the weight method as a percentage of bone dry weight^30,79^. The contents of calcium (Ca) and phosphorus (P) in the bone ash was determined using ICP-OES spectrometry on an Optima 7300 DV apparatus (Perkin Elmer, Boston, MA, USA) after prior mineralization of bone in nitric acid. The Ca and P content in samples were expressed in mg in 1 g of crude ash^80^.

### Statistical analysis

Data were analyzed using Statistica 13.0 (TIBCO Software Inc., Palo Alto, USA) software package. All results are expressed as least-square mean (LSM) with standard error (SE). Differences among the means were tested with a two-way ANOVA (with group of birds—capons and cockerels and age as factors) and post hoc Tukey’s test as the correction for multiple comparisons. The normality of data was checked using the Shapiro–Wilk test, while the equality of variance using Brown-Forsythe test. If there was a lack of normal distribution and/or unequal variance of data, the log transformation was applied. When the data still did not meet the assumptions for the parametric test, the Kruskal–Wallis test was used. A probability of *P* < 0.05 was considered statistically significant.

## Data Availability

The data presented in this study are available on request from the corresponding author.

## Abbreviations

ε_y_: Yield strain
ε_f_: Ultimate strain
σ_y_: Yield stress
σ_f_: Ultimate stress
A: Cortical cross-sectional area
BMC: Bone mineral content
BMD: Bone mineral density
Ca: Calcium
CI: Cortical index
E: Young modulus
F_el_: Yield load
F_max_: Ultimate load
H: Horizontal external diameter
h: Horizontal internal diameter
I_x_: Second (cross-sectional) moment of inertia
LSM: Least-square mean
M: Bending moment
MRWT: Mean relative wall thickness
P: Phosphorus
R_g_: Moment of gyration
S: Stiffness
SE: Standard error
u: Toughness modulus
V: Vertical external diameter
v: Vertical internal diameter
W_el_: Elastic energy
W_max_: Work to fracture
